# Endothelial Lipase: A New Risk Factor for Atherosclerosis?

**DOI:** 10.1371/journal.pmed.0030054

**Published:** 2005-12-20

Atherosclerosis, or hardening of the arteries, is the most common cause of death in industrialized countries. The formation in the arteries of deposits or plaques containing cholesterol, calcium, and other materials directly causes heart attacks, strokes, and peripheral circulatory problems by blocking the blood flow to the heart, brain, or limbs, respectively. In addition, plaque rupture causes the formation of blood clots, which can block arterial blood flow anywhere in the body.

Major risk factors for atherosclerosis include high blood pressure, obesity, smoking, and levels of certain cholesterol-containing particles in the blood stream. Cholesterol is carried around the body by particles called lipoproteins, molecules that include both lipids (fats) and proteins. Low-density lipoprotein (LDL) particles transport cholesterol to the arteries and other tissues that take up lipids. High blood concentrations of LDL particles are associated with high risk of atherosclerosis; the cholesterol carried by LDL (LDL-C) is “bad” cholesterol. By contrast, high concentrations of high-density lipoprotein (HDL) particles protect against cardiovascular disease by removing cholesterol from the arteries and taking it to the liver for excretion. Consequently, cholesterol carried by HDL (HDL-C) is “good” cholesterol, and individuals with high plasma concentrations of HDL-C and low concentrations of LDL-C are at lowest risk for cardiovascular disease.

Enzymes known as lipases modulate lipoprotein metabolism, and the activity of two of them—lipoprotein lipase and hepatic lipase—is known to affect the risk of atherosclerosis. Karen Badellino and colleagues are investigating whether a third lipase—endothelial lipase (EL)—also affects atherosclerosis risk in people, since this enzyme digests HDL and affects the development of atherosclerosis in mouse models. [Fig pmed-0030054-g001]

**Figure pmed-0030054-g001:**
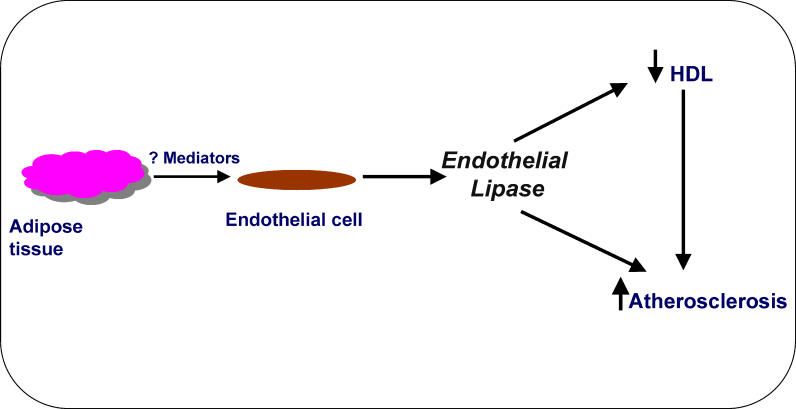
Endothelial lipase and atherosclerosis

Badellino and colleagues have used a new immunoassay to measure plasma EL concentrations in 858 unrelated people enrolled in the Study of Inherited Risk of Atherosclerosis, a cross-sectional study designed to investigate biomarkers and genetic factors associated with coronary atherosclerosis. Participants, who were just examined once rather than followed over time, as happens in a prospective, longitudinal study, had a family history of premature coronary artery disease (CAD) but were asymptomatic and had no other major risk factors for CAD. Because EL is normally bound tightly to heparin and is largely unavailable for immunoassay, the authors also measured EL concentrations in 510 individuals after heparin treatment to get an accurate measure of total EL mass. They then checked whether pre and postheparin EL levels were linked in any way to the concentrations of specific lipoproteins, components of the metabolic syndrome (a syndrome associated with cardiovascular disease that includes high blood pressure and obesity), or coronary arterial calcification, a noninvasive measure of early coronary atherosclerosis.

The researchers found that although the average postheparin concentration of EL was about three times the average preheparin concentration, pre and postheparin levels in individuals were strongly correlated. This result suggests that measurements of EL levels in untreated individuals will provide a good indication of the total vascular expression of EL for future studies. Then, they discovered that EL plasma levels both before and after heparin treatment correlated positively with components of the metabolic syndrome. Furthermore, as in mice, high levels of EL correlated with low levels of HDL-C, or “good” cholesterol. This last inverse association was small but statistically significant, as was the positive association between EL concentrations and signs of early atherosclerosis, even after taking into account other risk factors for atherosclerosis.

Overall, the authors suggest that EL is a proatherogenic factor in people, particularly those with metabolic syndrome. EL concentrations, they suggest, may modulate the composition of lipoproteins in the blood and thus atherosclerosis risk. But, they note, only prospective, longitudinal studies in which EL concentrations are correlated with subsequent heart attacks, strokes, and other cardiovascular problems can show whether EL concentration really is a risk factor for atherosclerosis, and thus whether interventions designed to reduce EL activity can prevent atherosclerosis.

